# Intrinsically dominant conformational diversity in PDZ1 within the tandem PDZ1–PDZ2 of human syntenin‐1 underlied by crystal structures

**DOI:** 10.1002/pro.70607

**Published:** 2026-05-05

**Authors:** Natsuno Ando, Yuya Hanazono, Koya Sakuma, Nobutaka Numoto, Ryusei Hamajima, Takeshi Tenno, Atsunori Oshima, Nobutoshi Ito, Hidekazu Hiroaki

**Affiliations:** ^1^ Graduate School of Pharmaceutical Sciences Nagoya University Nagoya Aichi Japan; ^2^ Laboratory of Structural Biology, Medical Research Laboratory Institute of Integrated Research, Institute of Science Bunkyo‐ku Tokyo Japan; ^3^ Cellular and Structural Physiology Institute (CeSPI) Nagoya University Nagoya Aichi Japan; ^4^ International Center for Structural Biology, Research Institute for Interdisciplinary Science Okayama University Okayama Japan; ^5^ BeCellBar, LLC Nagoya Aichi Japan; ^6^ Institute for Glyco‐core Research (iGCORE) Tokai National Higher Education and Research System Nagoya Aichi Japan; ^7^ Center for One Medicine Innovative Translational Research; COMIT Nagoya University Nagoya Aichi Japan

**Keywords:** conformational diversity, molecular dynamics simulation, protein–protein interactions, structural plasticity, syntenin‐1, tandem PDZ domains, x‐ray crystallography

## Abstract

The intrinsic dynamic asymmetry between homologous PDZ domains in multidomain scaffold proteins offers insight into how they achieve multivalent partner recognition. Through a systematic x‐ray crystallographic analysis of tandem PDZ1–PDZ2 domains in human syntenin‐1 (SDCBP/MDA‐9), we solved nine high‐resolution structures and uncovered fundamental differences in conformational plasticity between these sequentially similar domains. Pairwise root mean square deviation (RMSD) analysis of 20 PDZ1 structures across multiple crystal forms revealed substantial structural variability concentrated in the Lys119‐Ile125 and Ala181‐Glu184 loops—key regions governing ligand specificity within PDZ1's binding cleft—whereas PDZ2 maintained remarkable structural conservation, indicating divergent evolutionary constraints on these tandem domains. Comparative analysis of isotropic B‐factors and multistructure RMSD highlighted the limitations of B‐factors alone and emphasized the value of multistructure comparisons for mapping dynamic landscapes. Molecular dynamics (MD) simulations implemented through GROMACS corroborate the crystallographic observations, showing elevated residue‐specific fluctuation values in PDZ1's ligand‐binding interface compared to analogous PDZ2 regions, and steady‐state heteronuclear NOE measurements support enhanced loop flexibility in PDZ1 relative to PDZ2. Together, these findings indicate that PDZ1's conformational diversity represents an inherent biophysical property rather than a crystallographic artifact, suggest a functional division of labor in which PDZ1's structural plasticity enables broad ligand recognition via conformational selection while PDZ2's rigid architecture stabilizes the tandem domain arrangement, and provide an atomic‐level framework for developing domain‐selective therapeutics targeting syntenin‐1 in cancer, viral infection, and neurodevelopmental disorders.

## 1 INTRODUCTION

Syntenin‐1 (also known as SDCBP/MDA‐9) is a 32 kDa cytosolic scaffold protein that serves as either an adaptor or a critical hub in cellular signaling and trafficking networks (Grootjans et al., 1997). The architecture of syntenin‐1 features two postsynaptic density‐95/discs large/zonula occludens‐1 (PDZ) domains in tandem (PDZ1 and PDZ2) flanked by N‐ and C‐terminal regions, which enable it to act as a versatile protein–protein interaction hub. While syntenin‐1 has been extensively studied in the context of its role in exosome biogenesis through interactions with syndecans and ALIX (Baietti et al., 2012), emerging evidence highlights its other broader functions, including synaptic regulation (Hirbec et al., 2005), cytoskeletal dynamics (Zimmermann et al., 2001), receptor trafficking (Zimmermann et al., 2005), and viral entry (Gordón‐Alonso et al., 2012). These activities are linked to its divergent subcellular localization, which spreads from the plasma membrane, focal adhesions, and endosomal compartments to the early secretory pathway.

A key feature of syntenin‐1 is its ability to coordinate signaling complexes via PDZ domain–mediated interactions. The PDZ tandem binds diverse partners, including Frizzled receptors (Egea‐Jimenez et al., 2016), phosphatidylinositol 4,5‐bisphosphate (PIP2) (Zimmermann et al., 2002), and viral proteins (Jimenez‐Guardeño et al., 2014), through cooperative mechanisms that enhance binding avidity. For example, PDZ2 engages in tripartite interactions with the Frizzled 7 C‐terminus and PIP2, leveraging adjacent binding pockets to stabilize membrane‐associated signaling platforms (Egea‐Jimenez et al., 2016). Such interactions underpin syntenin‐1's role in noncanonical Wnt signaling and syndecan recycling—processes that are critical for cell polarity and growth factor responses (Luyten et al., 2008).

The involvement of syntenin‐1 in many important biological processes highlights it as a target for drug discovery. Thus, the atomic‐resolution structures of syntenin‐1's PDZ domains are critical for rational drug design. The x‐ray crystal structure of PDZ1 bound to the inhibitor KSL‐128018 (PDB code: 6AK2) reveals a noncanonical binding mode involving residues beyond the canonical groove, including Trp‐4 and Chg‐2, which engage in hydrogen bonds and hydrophobic interactions (Kang et al., 2003). Similarly, PDZ2's PIP2‐binding pocket, which is adjacent to the peptide groove, is stabilized by water‐mediated networks (Zimmermann et al., 2002). These structural details may explain how syntenin‐1 achieves high‐affinity interactions with diverse partners and highlight targetable regions for disrupting oncogenic signaling.

Accordingly, the extended binding interfaces and hydration networks between PDZ1 and PDZ2 position syntenin‐1 as a promising therapeutic target (Das & Fisher, 2025; Kegelman et al., 2015). Inhibitors such as KSL‐128114, designed to leverage these structural features, demonstrate nanomolar affinity, metabolic stability, and efficacy in glioblastoma models (Haugaard‐Kedström et al., 2021). Targeting syntenin‐1's PDZ‐mediated interactions with syndecans, Frizzled receptors, or viral proteins (e.g., SARS‐CoV‐2 E protein) could mitigate cancer metastasis, neurodevelopmental disorders, and viral pathogenesis, respectively (Boukerche et al., 2005; Gordón‐Alonso et al., 2012; Kegelman et al., 2013). Thus, high‐resolution structural data of individual PDZ domains may further enable the development of domain‐selective inhibitors to modulate syntenin‐1's multifunctional role in disease.

Initially, we aimed to resolve the drug–PDZ1 complex structure for anthranilic acid derivatives (NPL compounds), analogs of Disheveled PDZ inhibitors (Hori et al., 2018). While cocrystallization attempts with these compounds were unsuccessful, we determined 10 high‐resolution apo‐form crystal structures of the syntenin‐1 PDZ1–PDZ2 tandem, including one crystal of the PDZ1–PDZ2 tandem complexed with compound E5 (PDZ2i) (Hoffer et al., 2023). Structural analysis revealed significant conformational variation in PDZ1's canonical ligand‐binding pocket, in contrast with PDZ2's static architecture. Here, we evaluate conformational divergence across 20 PDZ1 structures from the 10 independent crystals and compare them to PDZ2. GROMACS‐based molecular dynamics (MD) simulations (van der Spoel et al., 2005) of isolated PDZ1 and PDZ2 domains further highlighted intrinsic flexibility in PDZ1's Lys119–Ile125 and Ala181–Glu184 loops.

## 2 RESULTS AND DISCUSSION

As described above, we initially attempted to obtain the complex structure between the syntenin‐1 PDZ1 and anthranilic acid PDZ‐domain inhibitor derivatives. These compounds were analogs of Disheveled PDZ inhibitors (Hori et al., 2018) (Supplementary Figure S1) and demonstrated binding to human syntenin‐1 PDZ1 but not PDZ2 in our preliminary study. Although we failed to obtain any one of the cocrystals, we succeeded in determining nine high‐resolution apo‐form crystal structures of the syntenin‐1 PDZ1–PDZ2 tandem. Additionally, we succeeded in cocrystallizing the PDZ1–PDZ2 tandem complexed with compound E5 (PDZ2i), which is a reported PDZ2 inhibitor (Hoffer et al., 2023). All the PDZ1–PDZ2 tandem structures resemble the reported syntenin1 PDZ1–PDZ2 crystals, such as PDB code: 8BLU. However, we eventually found that, interestingly, only one PDZ1 domain of chain B of the dimeric crystal architecture showed high structural diversity during the refinement process of these 10 crystals. In detail, apparently large conformational variation in PDZ1's loops surrounding the canonical ligand‐binding pocket was found, whereas that of PDZ2's seemed static and not mobile.

**FIGURE 1:**
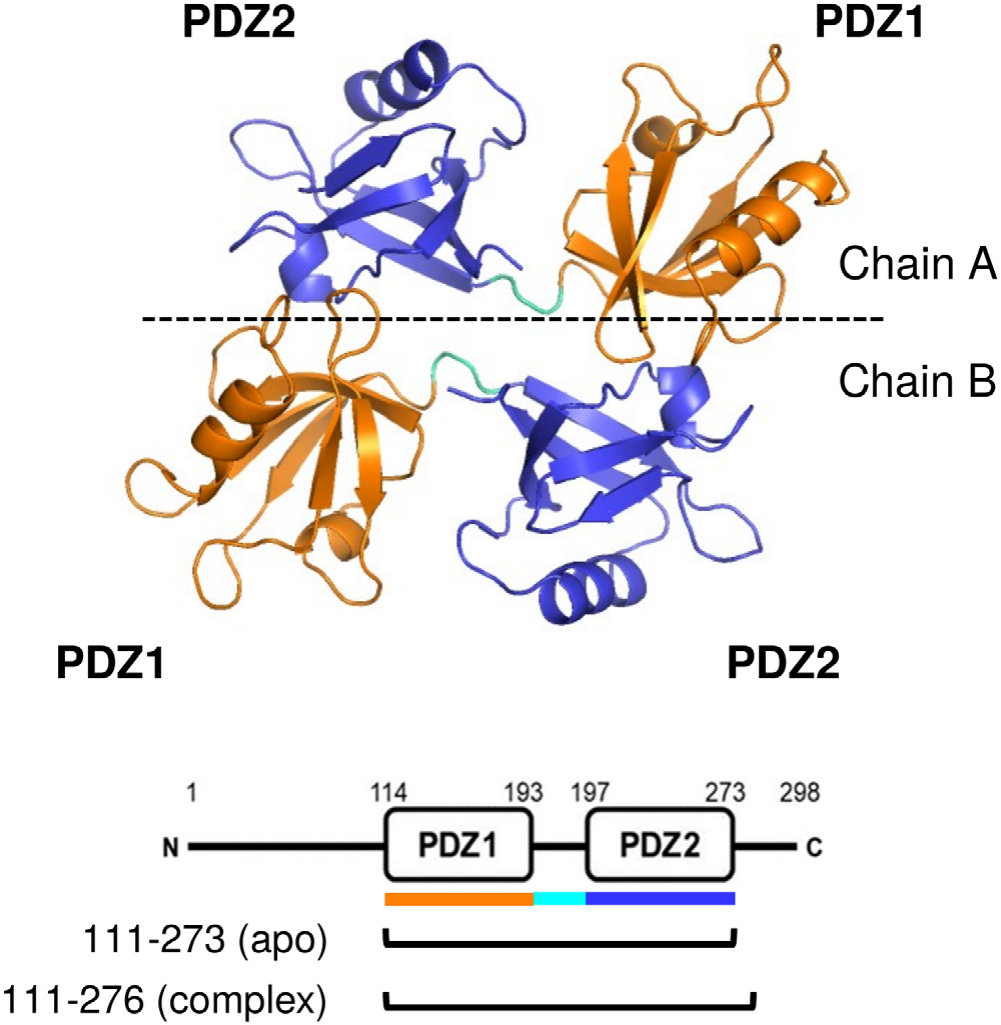
A representative crystal structure (top, crystal entry s1, PDB ID:9VA6) and domain architecture of apo‐syntenin‐1 (bottom). The crystal structure contains two molecules of syntenin‐1. The PDZ1 domain is assigned to Glu114 to Arg193 (orange), and the PDZ2 domain is assigned to Arg197 to Phe273 (blue). A 163‐amino acid construct (residues 111–273) was used to determine the structure of apo‐syntenin‐1, while a 166‐amino acid construct (residues 111–276) was used to determine the structure of the syntenin‐1‐compound complex.

**FIGURE 2:**
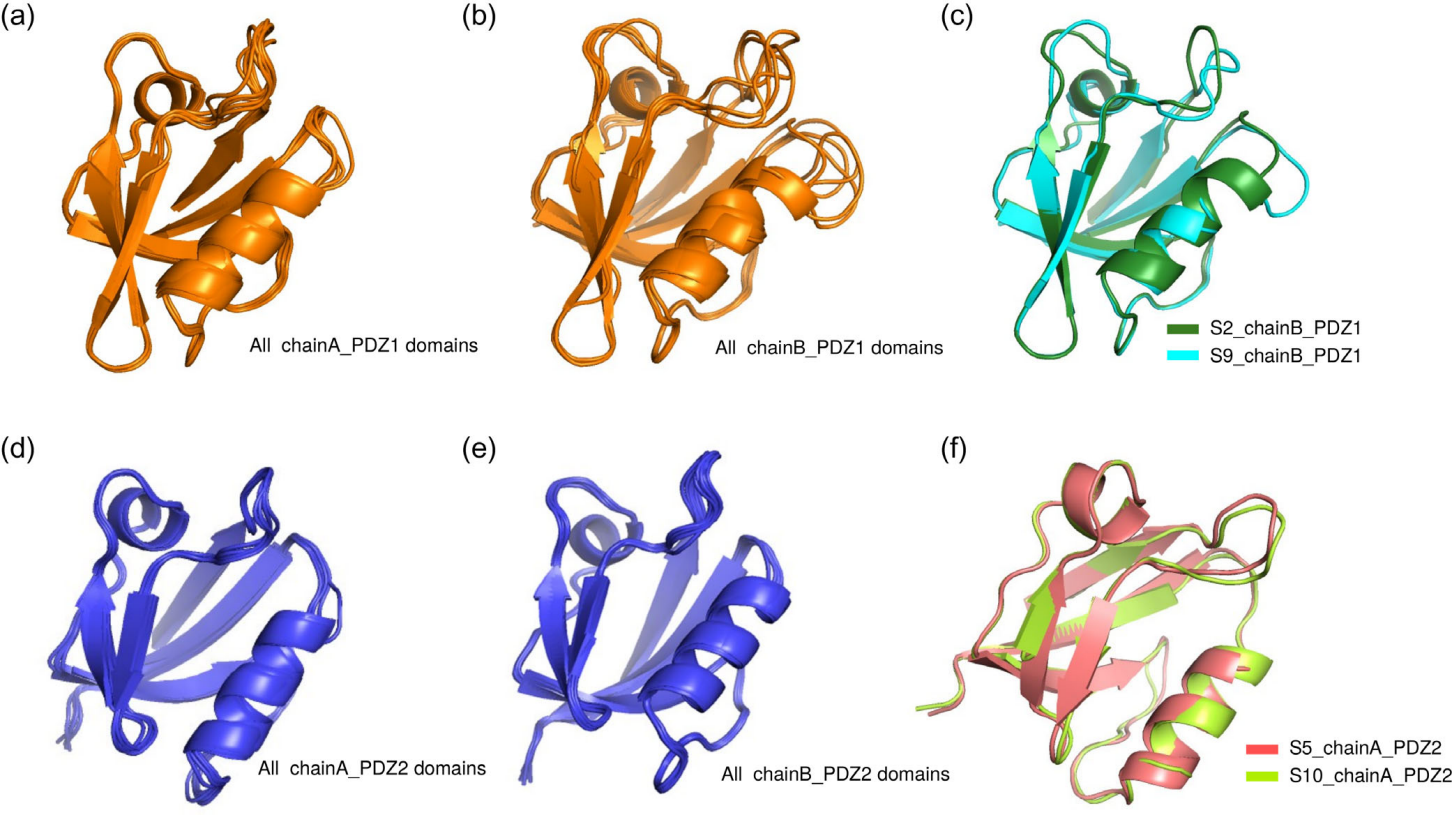
Comparison by superposition between each PDZ domain. (a) Comparison of all nine structures of the apo PDZ1 domain of chain A. (b) Comparison of all nine structures of the apo PDZ1 domain of chain B. (c) Comparison of the pairs with highest pairwise RMSD among PDZ1 domain of chain B. The S2_chainB_PDZ1 structure is highlighted as green, and the S9_chainB_PDZ1 structure is highlighted as cyan. (d) Comparison of all nine structures of the apo PDZ2 domain of chain A. (e) Comparison of all nine structures of the apo PDZ2 domain of chain B. In contrast to PDZ1, rigid structures were observed in both chain A and chain B in PDZ2. (f) Comparison of the bound and unbound structures of the PDZ2 domain. The S5_chainA_PDZ2 structure (unbound structure) is highlighted as orange, and the S10_chainA_PDZ2 structure (bound structure) is highlighted as yellow.

**FIGURE 3:**
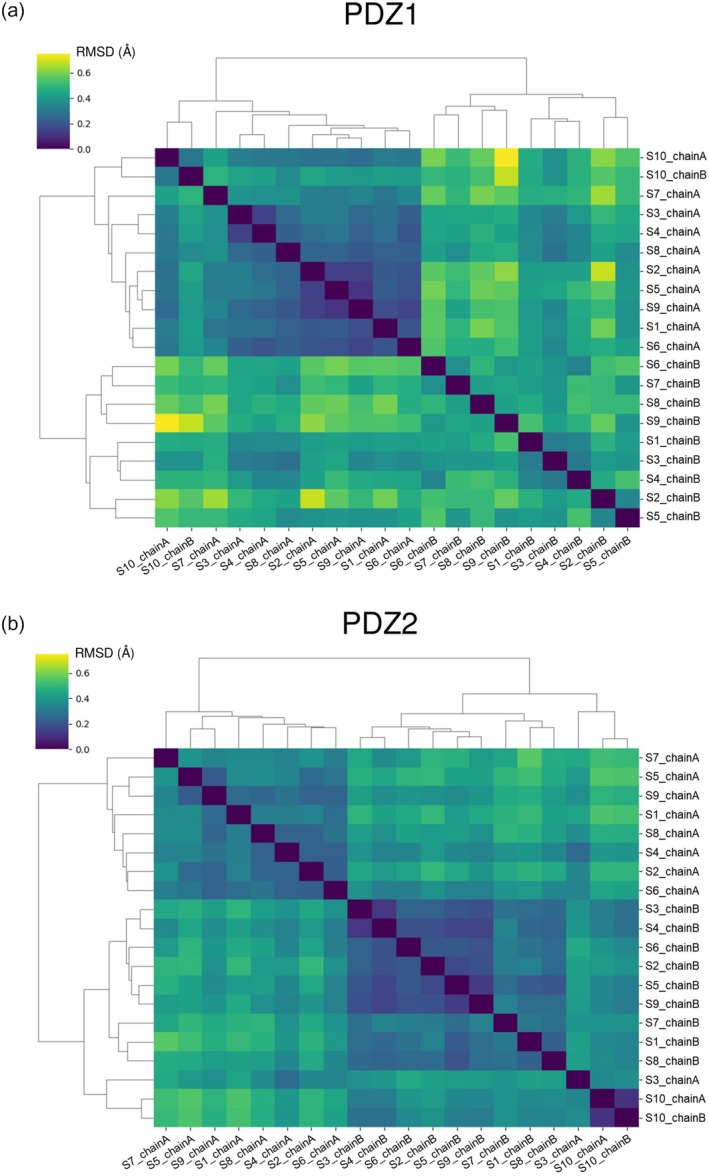
Cluster analysis of syntenin‐1 PDZ domains. The structures of PDZ domains were extracted one by one from nine apo structures and one complex structure, and the structures of 20 PDZ domains were obtained. Pairwise RMSD was calculated for all pairs produced from the 20 PDZ domains. Pairwise RMSD levels were classified from yellow to dark blue, and the similarity between each pair is represented in a heatmap. Based on similarity, a cluster analysis was performed, and the results are expressed in a cluster dendrogram. (a) Cluster analysis of PDZ1 domains. Major clusters were formed in chain A and chain B. (b) Cluster analysis of PDZ2 domains. Major clusters were formed in apo chain A and apo chain B. A minor cluster was formed by S3 chain A and S10 structures.

To gain comprehensive insights into the dynamic structural changes of syntenin‐1, we conducted a statistical comparison of the crystal structures obtained. As depicted in Figure 1, syntenin‐1 molecules form dimeric assemblies within the crystal lattice, resulting in each asymmetric unit containing four PDZ domains (two PDZ1 and two PDZ2 domains from two chains). From a total of 10 determined crystal structures, we obtained 20 structures for the PDZ1 domain and 20 structures for the PDZ2 domain. The PDZ1 domain encompasses residues Glu114 to Arg193 (colored orange), while the PDZ2 domain spans residues Arg197 to Phe273 (colored blue). The apo‐syntenin‐1 structure was determined using a 163‐amino acid construct, whereas a 166‐amino acid construct was utilized to determine the structure of the syntenin‐1‐compound complex. Then, a comparative analysis of these 10 PDZ1–PDZ2 tandem structures revealed significant conformational heterogeneity exclusively within the PDZ1 domain of chain B. In contrast, the PDZ1 domain of chain A and both PDZ2 domains (chains A and B) exhibited structural conservation, as evidenced by superimposition analyses (Figure 2a,b,d,e). To quantify this observed divergence, we conducted statistical evaluations of the root mean square deviation (RMSD) of backbone Ca positions. Initially, we classified these crystal structures based on their structural similarity. Pairwise RMSD values were computed for all possible pairs derived from the 20 PDZ1 and 20 PDZ2 domain structures. The resulting two‐dimensional data matrix of pairwise RMSD was subjected to hierarchical clustering in both rows and columns. The data were sorted by closely related structural clusters in proximity. A heatmap was then generated, with color coding indicating the level of pairwise RMSD (Figure 3).

For the PDZ1 domain, major clusters were observed to form between chain A/chain B pairs (Figure 3a). It should be noted that the dominant structural difference between chain A and chain B probably arises from crystal‐packing contacts, and we therefore concluded that the overall structural difference observed between PDZ1_chain_A and PDZ1_chain_B does not reflect the intrinsically dynamic nature of PDZ1. In contrast, relatively large structural fluctuations were observed among the PDZ1 domains of chain B/chain B pairs, suggesting that the intrinsic structural flexibility of the syntenin‐1 PDZ1 domain becomes apparent in this context. Among this cluster, however, apo6 and apo7, which were crystallized without compounds, fell into the same structural subcluster, separate from the other subclusters that were crystallized with NPL inhibitors, so we could not rule out that the presence of inhibitors in crystallizing conditions exerts some influence on the conformational variations of PDZ1. Conversely, PDZ1 of chain A/chain A pairs did not exhibit remarkable pairwise RMSD values, indicating generally similar structures across these instances (Figure 3a), and the presence or absence of a compound in the crystallization condition did not influence the clustering of chain A/chain A pairs, suggesting that the low structural diversity was primarily due to the restrictions imposed by crystal contacts rather than the presence of PDZ inhibitors. In addition, to visualize the differences between the clusters more quantitatively, we calculated the inter‐cluster distances in the hierarchical clustering results (Supplementary Figure S5).

Next, we analyzed the conformational variations of PDZ2 domains, which also exhibited two dominant clusters based on the difference between chain A and chain B (Figure 3b). However, unlike PDZ1, the hierarchical clustering analysis of the PDZ2 structure succeeded in separating structures of apo3_chain A and both chain A and chain B of the complex structures into distinct major clusters. This result can be explained by the binding of the inhibitor PDZ2i, which may fix the loop conformation. However, we observed that the apo3_chain A structure was very similar to that of the complex structures. Thus, we expected that a “conformational selection” mechanism may work rather than “induced fit” upon PDZ2i binding to PDZ2 of syntenin‐1. Accordingly, when comparing the structural differences between PDZ1 and PDZ2, PDZ1 consistently exhibited higher pairwise RMSD values than PDZ2. This observation strongly led us to hypothesize that PDZ1 possesses greater structural flexibility than PDZ2.

Accordingly, we visualized the selected pairs of PDZ domains and structural clusters by superimposing (Figure 2). The apo2_chainB_PDZ1/apo9_chainB_PDZ1 pair displayed the largest pairwise RMSD value among all PDZ1 pairs (Figure 2c). The PDZ1 domain demonstrated diverse structures in the loops surrounding the canonical ligand‐binding pocket. As mentioned previously, greater structural polymorphism was consistently observed in the PDZ1 domains of chain B compared to those of chain A when all obtained PDZ1 structures within chain A and chain B had compared each other, respectively (Figure 2a,b). In contrast, comparisons of PDZ2 domains within chain A and chain B (Figure 2d,e) revealed no significant structural polymorphism, suggesting a rigid structure for PDZ2. Furthermore, a comparison between the bound and unbound structures of the PDZ2 domain (Figure 2f) exhibited only a small structural difference in a loop region analogous to where polymorphism was observed in the PDZ1 domain.

**FIGURE 4:**
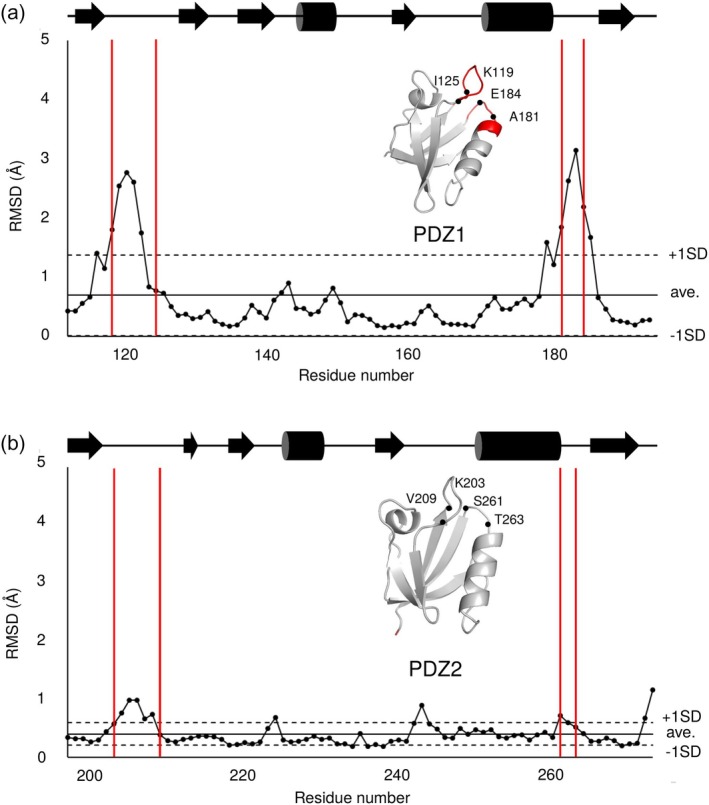
Average pairwise root mean square deviation (RMSD) for each residue calculated by pairwise comparison of crystal structures. Highlighted areas (red) are the sites of molecular fluctuations in the PDZ1 domain. (a) Average pairwise RMSD for each residue in the PDZ1 domain. (b) Average pairwise RMSD for each residue in the PDZ2 domain. The diagrams of the secondary structures are shown at the top of the panels. The high‐RMSD regions (>1.0 Å) on PDZ domain structures were highlighted as red.

To analyze this structural diversity in detail, we calculated the RMSD for each residue within the syntenin‐1 PDZ domains. For all PDZ domain pairs depicted in Figure 2, the pairwise RMSD for each residue was calculated, and their average values were plotted against the residue numbers (Figure 4). For PDZ1, locally high average pairwise RMSD values were observed in the Lys119–Ile125 loop and the Ala181–Glu184 loop, which surround the canonical ligand‐binding pocket (Figure 4a), suggesting that these regions are intrinsically flexible. Additionally, minor peaks were observed at Ala143 and Leu149. In contrast, for PDZ2, although peaks were present in the corresponding loops, they did not reach the high average pairwise RMSD values observed for PDZ1 (Figure 4b); small peaks were also observed at Asp224 and Gly243. In addition, for the 9V90 complex structure, the overall *R*_merge_ value was low, indicating high crystal quality, whereas several RSRZ outliers (indicating local model–density discrepancies) were detected in the PDZ1 loop region, which may reflect genuine conformational heterogeneity. Accordingly, we further confirmed the higher structural diversity of PDZ1 by analyzing all available syntenin‐1 PDZ1–PDZ2 tandem crystal structures in the PDB (Supplementary Figure S2). Overall, the crystal structure analysis clearly indicates that PDZ1 exhibits higher structural flexibility than PDZ2, particularly in these loop regions.

To further examine the relationship between structural diversity and local disorder, we compared B‐factor profiles across all PDZ1 and PDZ2 structures (Supplementary Figure S6). Specifically, we plotted the B‐factor values for the structure with the highest B‐factors, the average values across all structures, and the structure with the lowest B‐factors. This analysis revealed a clear tendency for higher B‐factor values in the loop regions of PDZ1 compared to PDZ2, consistent with the enhanced flexibility inferred from RMSD and RMSF analyses. Additionally, relatively high B‐factor values were observed in the Ser145–Gly151 segment of PDZ1, suggesting that this region also exhibits elevated local mobility.

**FIGURE 5:**
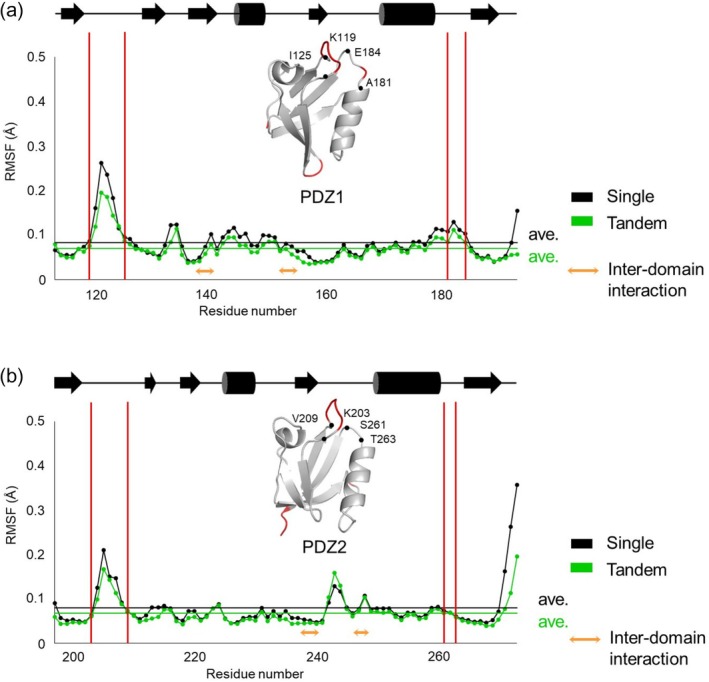
Root mean square fluctuation (RMSF) for each residue calculated by MD calculations on the single PDZ1/PDZ2 domain (black) and the PDZ1‐PDZ2 tandem structure (green). The S6_chain A structure was used for simulation. Highlighted areas show molecular fluctuations in the PDZ1 domain. (a) RMSF for each residue in the PDZ1 domain. (b) RMSF for each residue in the PDZ2 domain. The diagrams of the secondary structures are shown at the top of the panels. The high‐RMSF regions (>0.12 Å) on PDZ domain structures were highlighted as red. Inter‐domain contact residues in PDZ1 (Val138–Val141, Leu152–Gly155) and PDZ2 (Asn237–Glu240, Val246–Gly248) were highlighted as orange.

**FIGURE 6:**
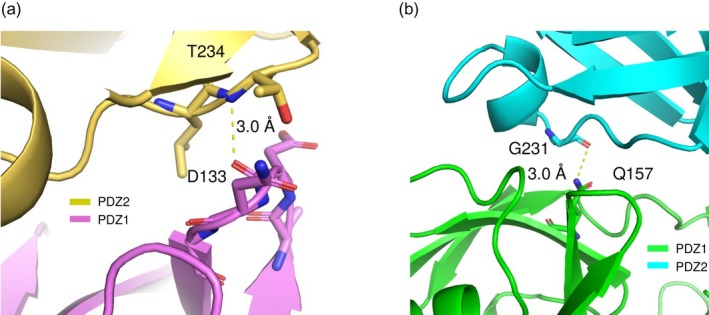
The residues that exhibited a high RMSF in MD simulations with low structural diversity in the 10 crystal structures. (a) Around Ile132 to Gly135 in PDZ1. (b) Around Asn230 to Leu232 in PDZ2.

The structural heterogeneity observed in PDZ1 loop regions across the 10 crystallographic datasets prompted the hypothesis that these loops exhibit inherent conformational flexibility in solution, a property potentially linked to syntenin‐1's multifunctional roles in cellular processes. To evaluate this hypothesis, we conducted MD simulations of both the isolated human syntenin‐1 PDZ1 and PDZ2 domains and the tandem PDZ1–PDZ2 construct using the GROMACS software package (Figure 5 and Supplementary Figure S3, starting from the different chain of the same crystal S6 chain A and B, respectively). Comparative analysis in both systems revealed enhanced flexibility in the PDZ1 loop regions relative to PDZ2, consistent with crystallographic observations. Strikingly, the simulations also identified two previously uncharacterized regions of elevated mobility: residues Ile132–Gly135 in PDZ1 (region i) and residues Asn230–Leu232 in PDZ2 (region ii). Both regions were localized to the PDZ1–PDZ2 interdomain interface (Figure 6), where more than 80% of their constituent residues participate in interdomain contacts. In detail, the main chain and side chain of Asp133 in region i formed hydrogen bonds with Thr234 in PDZ2, whereas Gly231 in region ii formed a hydrogen bond with Gln157 in PDZ1 (Figure 6a,b). Thus, these regions may possess additional intrinsic flexibility, even though they adopt relatively fixed conformations within the tandem PDZ1–PDZ2 architecture of human syntenin‐1. This interpretation is partly supported by the observation that the RMSF values for interdomain contact residues in PDZ1 (Val138–Val141, Leu152–Gly155) and PDZ2 (Asn237–Glu240, Val246–Gly248) showed no substantial differences between the single‐domain and tandem simulations.

Finally, we assessed whether the loop heterogeneity observed in the crystal structures is preserved in solution using steady‐state heteronuclear NOE experiments (Supplementary Figure S4). In PDZ1, the Ala181–Glu184 loop showed reduced NOE intensity, consistent with enhanced local flexibility, whereas the Lys119–Ile125 loop exhibited only modest changes despite its pronounced crystallographic heterogeneity, suggesting that conformational exchange in this region may occur on a timescale slower than that optimally probed by the heteronuclear NOE measurement. In PDZ2, only the Lys203–Val209 loop displayed a modest decrease in NOE intensity, and no clear change was detected at Ser261–Thr263. Taken together, these NMR data partly support the conclusion that PDZ1, particularly the Ala181–Glu184 loop (and likely also the Lys119–Ile125 loop), is more flexible in solution than PDZ2, in agreement with the crystallographic and MD analyses.

Overall, our crystallographic, MD, and NMR data suggest that enhanced flexibility in the PDZ1 loop regions is a genuine and functionally relevant property of syntenin‐1. In line with this notion, the GABA transporter GAT1 has been recently identified as a physiological binding partner of syntenin‐1 PDZ1 (Koščová et al., 2025). In that study, residues within the Lys119–Ile125 loop (Asp120, Asp122, Lys124) and nearby positions (Arg128, His175) were proposed to make key electrostatic and aromatic contacts with the GAT1 C‐terminal tail, supporting the idea that this flexible loop contributes directly to ligand recognition. Related features have been described for other scaffold PDZ systems (Fujiwara et al., 2015; Mendes et al., 2020; Murciano‐Calles, 2020): PSD95‐PDZ3 populates multiple intermediate conformational states and displays notable structural plasticity, and GRASP proteins, which also contain a PDZ tandem, exhibit cooperative interdomain motions that can influence partner‐binding specificity. These observations place syntenin‐1 PDZ1 within a broader group of dynamically tuned PDZ scaffolds, while the pronounced asymmetry between PDZ1 and PDZ2 in syntenin‐1 appears to be a particularly interesting example compared with the more balanced behavior often reported for other PDZ tandems.

## 3 CONCLUSION

This study provides compelling evidence that PDZ1 in human syntenin‐1 exhibits inherent conformational diversity compared to PDZ2's rigid architecture despite their sequential similarity. Through extensive crystallographic analysis and MD simulations, we demonstrate that PDZ1's structural plasticity is concentrated in key ligand‐binding loops, suggesting a conformational selection mechanism for diverse partner recognition. In contrast, PDZ2 exhibits remarkable structural conservation, indicating distinct evolutionary constraints on these tandem domains. The observed dynamic asymmetry explains syntenin‐1's versatility in cellular signaling pathways and offers a rational foundation for developing domain‐selective inhibitors targeting cancer metastasis, viral pathogenesis, and neurodevelopmental disorders. Our findings highlight the importance of multistructure comparisons and hierarchical analysis for accurately mapping protein dynamic landscapes beyond what B‐factors alone can reveal.

## 4 METHODS

### 4.1 Expression and purification of protein samples

The DNA fragment encoding the human syntenin‐1 PDZ tandem (residues 113–273 and 113–276, see Figure 1) was amplified via polymerase chain reaction (PCR) and subsequently cloned into a glutathione‐S‐transferase (GST) fusion expression vector, which included a PreScission™ Protease (Cytiva, Tokyo, Japan) cleavage site. Protein expression was induced by the addition of 1 mM isopropyl *β*‐D‐1‐thiogalactopyranoside (IPTG) in *Escherichia coli* BL21‐CodonPlus (DE3)‐RIL Competent Cells (Agilent Technologies Japan, Ltd., Tokyo, Japan). The PDZ tandem was expressed in LB medium, and cell pellets were subsequently lysed by sonication. The lysate was clarified through centrifugation and purified by affinity chromatography using COSMOSIL® GST Accept (nacalai tesque, Inc., Kyoto, Japan) in a buffer containing 50 mM Tris–HCl (pH 7.5), 150 mM NaCl, and 1 mM EDTA. The GST‐tagged protein was then subjected to cleavage using PreScission™ Protease at 4°C. After complete digestion, the cleaved PDZ tandem was separated from GST and further purified using gel filtration chromatography using HiLoad™ 26/600 Superdex™ 75 prep grade (Cytiva). The protein‐containing elution was subsequently dialyzed in a buffer composed of 25 mM HEPES (pH 7.4), 150 mM NaCl, and 1 mM DTT.

### 4.2 Crystallization and data collection

Crystallization experiments were conducted using the sitting drop vapor diffusion method at 20°C. The specific crystallization conditions for the apo‐syntenin‐1 PDZ protein are detailed below. In this study, we initially intended to obtain the crystals of the complex between the PDZ tandem and the anthranilic PDZ inhibitors NPL1010, 3005, 3026, and 3027 (Hori et al., 2018). The chemical structures of the compounds are summarized in Supplementary Figure S1.

- S1 (PDB code: 9VA6): Crystals were obtained from a solution containing 5 mM NPL3005, 0.2 M sodium chloride, 0.1 M HEPES pH 7.5, and 25% w/v polyethylene glycol (PEG) 3350. [Correction added on 20 May 2026, after first online publication: The PDB code “3TYS” has been corrected to “9VA6” in this version.]
- S2 (PDB code: 9VA9): Crystals were obtained from a solution containing 5 mM NPL3005, 0.2 M sodium formate, and 20% w/v PEG 3350.
- S3 (PDB code: 9VAC): Crystals were obtained from a solution containing 5 mM NPL3026, 0.2 M ammonium acetate, 0.1 M sodium citrate tribasic dihydrate pH 5.6, and 30% w/v PEG 4000.
- S4 (PDB code: 9VAD): Crystals were obtained from a solution containing 5 mM NPL3027, 0.2 M lithium sulfate monohydrate, 0.1 M Tris pH 8.5, and 25% w/v PEG 3350.
- S5 (PDB code: 9VAF): Crystals were obtained from a solution containing 5 mM PDZ1i, 0.2 M sodium chloride, 0.1 M BIS‐TRIS pH 5.5, and 25% w/v PEG 3350.
- S6 (PDB code: 9VAI): Crystals were obtained from a solution containing 0.2 M ammonium acetate, 0.1 M sodium acetate trihydrate pH 4.6, and 30% w/v PEG 4000.
- S7 (PDB code: 9VAL): Crystals were obtained from a solution containing 0.2 M sodium chloride, 0.1 M Tris pH 8.5, and 25% w/v PEG 3350.
- S8 (PDB code: 9VB9): Crystals were obtained from a solution containing 5 mM NPL1010, 0.1 M BIS‐TRIS pH 5.5, and 25% w/v PEG 3350.
- S9 (PDB code: 9VBB): Crystals were obtained from a solution containing 5 mM NPL1010, 0.2 M ammonium acetate, 0.1 M BIS‐TRIS pH 5.5, and 25% w/v PEG 3350.

The crystallization conditions for the syntenin‐1 PDZ protein–compound complex are outlined below:

- S10 (PDB code: 9V90): Cocrystallization was performed in a solution containing 0.5 mM PDZ2i, 0.4 M ammonium acetate, 0.1 M sodium acetate pH 4.6, and 20% PEG 3350.

All crystals were grown from a protein concentration of 6 mg/mL. Crystals were cryo‐protected by immersion in mother liquor supplemented with 10% (v/v) glycerol before freezing. Diffraction data for syntenin‐1 PDZ crystals were collected at the Photon Factory BL‐17A and processed using x‐ray detector software (Kabsch, 2010). The phase problem was solved by molecular replacement using the structure of PDB code: 8BLU as a template with the PHASER program (McCoy et al., 2007). The parameters of data collection and refinement statistics for all the crystal entries are summarized in Supplementary Table S1. The models were refined using Phenix (Adams et al., 2010). The figures were prepared using PyMOL software (http://www.pymol.org/).

### 4.3 Molecular dynamics

#### 4.3.1 Preparation of initial structures

For the simulations of isolated PDZ domains, the PDZ1 and PDZ2 domains were defined as residues 113–193 and 197–273, respectively, and extracted from chain A or chain B of the crystallographic asymmetric unit. To ensure consistent construction of simulation boxes for both PDZ1 and PDZ2 domains, the PDZ2 from chain A and domains from chain B were structurally aligned to the PDZ1 domain from chain A using the “align” command in PyMOL, and these superposed models were subsequently used as the initial structures. For the simulations of whole Syntenin‐1 structures, two intact chains, A and B, were extracted from the asymmetric unit, where chain B was superposed onto chain A similarly to the domain‐wise setup. For all six types of systems defined above, GROMACS (version: 2024.5) (van der Spoel et al., 2005) was employed as the MD engine, utilizing the charmm36‐jul2022.ff force field, a GROMACS adaptation of CHARMM36 (Huang et al., 2016). Throughout all simulation procedures, hydrogen atoms were constrained to their ideal geometry using the riding model, and a time step size of 2 fs was applied.

#### 4.3.2 Simulation system construction

The initial protein structures were embedded within a cubic box with 130.0 Å of edges for whole‐structure simulations, or a dodecahedron simulation box prepared with 11.0 Å margins for domain‐wise simulations, respectively. Prior to solvation, the system was energy‐minimized in a vacuum using the steepest descent algorithm for a maximum of 500,000 iterations to eliminate any steric clashes between atoms. The minimized model was then solvated with the TIP3P water model, whose geometry was provided as spc216. To achieve a neutral net charge, the system was ionized with 0.1 M equivalent NaCl‐derived ions, and the solvated system underwent a second round of energy minimization. Finally, the system was subjected to a very short NVT simulation (1 ps at 300 K), with harmonic restraints applied to all heavy atoms to ensure the absence of any severe residual steric clashes.

#### 4.3.3 Equilibration and production run

A 2 fs time step was consistently used in all MD simulations. The system was equilibrated through sequential 100 ps NVT and subsequent NPT simulations at 300 K and 1 bar, with heavy‐atom restraints for each trajectory. Different random seeds were used to generate the initial velocities for each independent run. Five independent 200 ns production runs were performed for each of the isolated PDZ1 domains, PDZ2 domains, and the whole Syntenin‐1 structures. The final 160 ns of each trajectory were subsequently extracted for detailed analysis.

#### 4.3.4 Fluctuation analysis

The root mean square fluctuation (RMSF) was computed for each C*α* atom from frames sampled every 100.0 ps from the final 160 ns of the 200 ns trajectories. These trajectory‐averaged residue‐wise RMSF values were then used to calculate their mean and standard deviation across the five independent runs. As for the analysis of whole‐structure simulations of Syntenin‐1, the domain definitions identical to the domain‐wise systems were applied to select residues to be superposed for the RMSFs calculation.

### 4.4 Heteronuclear NOE measurements

^15^N‐isotopically labeled PDZ1 and PDZ2 domains of human syntenin‐1 were prepared in M9 medium using ^15^N‐ammonium chloride as the sole nitrogen source. NMR measurements were carried out at 25°C on a Bruker Avance III 600 MHz spectrometer equipped with a cryogenic probe at Gifu University. For assignment of backbone ^1^H, ^13^C, and ^15^N resonances, HNCA, HNCACB, CBCA (CO) NH, HNCO, HN (CA)CO, and 2D ^1^H‐^15^N HSQC spectra at 25°C. For the heteronuclear NOE experiments, 0.4 mM 15 N‐labeled PDZ1 or PDZ2 was dissolved in 20 mM 3‐(N‐morpholino)‐propanesulfonic acid buffer (pH 6.8) containing 20 mM NaCl, 1 mM dithiothreitol, and 10% D_2_O. Steady‐state ^1^H–^15^N heteronuclear NOE spectra were acquired with and without 2 s proton saturation, peak intensities were integrated, and NOE ratios were calculated as *I*_sat_/*I*_unsat_ and plotted versus residue number, where *I*_sat_ and *I*_unsat_ are the signale intensities of ^1^H‐saturated and unsaturated conditions, respectively (Figure S4).

## AUTHOR CONTRIBUTIONS

*Conceptualization*: N.A. and H.H. *Investigation*: N.A., Y.H., K.S., R.H., N.N., and T.T. *Graphics, table and charts*: N.A. and K.S. *Data analysis*: N.A., Y.H., K.S., N.N., and T.T. *Resources*: Y.H., K.S., N.N., and T.T. *Writing and original draft preparation*: N.A. and H.H. *Writing*, *reviewing and editing*: N.I. and H.H. *Supervision*: N.I. and H.H. *Project administration*: H.H. Funding acquisition: H.H. All authors have read and agreed to the published version of the manuscript.

## FUNDING INFORMATION

This study was supported by Nanken‐Kyoten, Science Tokyo. This study was also supported by JSPS KAKENHI Grant Number [24K0214524] (Grant‐in‐Aid for Scientific Research (B)) to H.H., AMED Program for Innovative Drug and Medical Device Development against Emerging and Re‐emerging Infectious Diseases (22fk0108527) to H.H., and Astellas Foundation for Research on Metabolic Diseases (FY2021, COVID‐19 Special Grant) to H.H.

## CONFLICT OF INTEREST STATEMENT

Among the authors, T.T. and H.H. are the founders of a Nagoya University‐based spinoff startup company called BeCellBar, LLC. The remaining authors declare no conflicts of interest.

## Supporting information


**Supplementary Figure S1.** The chemical structures of the compounds used in crystallization experiments of human syntenin‐1 PDZ1‐PDZ2 tandem.
**Supplementary Figure S2.** Average pairwise root mean square deviation (RMSD) for each residue calculated by pairwise comparison of crystal structures in the PDB database. The reference PDB ID: 1N99, 1OBZ, 1V1T, 1W9E, 1W9O, 1W9Q, 1YBO, 4Z33, 6R9H, 6RLC, 8AAI, 8AAK, 8AAO, 8AAP. Highlighted areas (red) are the sites of molecular fluctuations in the PDZ1 domain. (A): Average pairwise RMSD for each residue in the PDZ1 domain. (B): Average pairwise RMSD for each residue in the PDZ2 domain. The diagrams of the secondary structures are shown at the top of the panels. The red lines indicate the two loop regions of each PDZ domains. The high‐RMSD regions (>1.0 Å) on PDZ domain structures were highlighted as red.
**Supplementary Figure S3.** Root mean square fluctuation (RMSF) for each residue calculated by MD calculations on the single PDZ1/PDZ2 domain (black) and the PDZ1–PDZ2 tandem structure (green). The S6_chain B structure was used for simulation. Highlighted areas show molecular fluctuations in the PDZ1 domain. (A): RMSF for each residue in the PDZ1 domain. (B): RMSF for each residue in the PDZ2 domain. The red lines indicate the two loop regions of each PDZ domains. The diagrams of the secondary structures are shown at the top of the panels. The high‐RMSF regions (>0.12 Å) on PDZ domain structures were highlighted as red.
**Supplementary Figure S4.** ^1^H–^15^N Hetero‐NOE measurements by solution NMR. (A): Peak intensity ratio for each residue in the PDZ1 domain. (B): Peak intensity ratio for each residue in the PDZ2 domain. The diagrams of the secondary structures are shown at the top of the panels. The red lines indicate the two loop regions of each PDZ domains. The low peak intensity ratio regions (<0.75) on PDZ domain structures were highlighted as red.
**Supplementary Figure S5.** Cluster analysis results with inter‐cluster distance values. (A): Cluster analysis of PDZ1 domains. (B): Cluster analysis of PDZ2 domains.
**Supplementary Figure S6.** The B‐factor values for the structure with the highest B‐factor (orange), the average value across all structures (black), and the structure with the lowest B‐factor (blue). (A): The B‐factor values for each residue in the PDZ1 domain. (B): The B‐factor values each residue in the PDZ2 domain. The diagrams of the secondary structures are shown at the top of the panels. The high B‐factor regions (>ave.) on PDZ domain structures were highlighted as red.
Supplementary Table S1.

## Data Availability

The crystallographic analysis datasets have been deposited to the Protein Data Bank. Other related data (MD trajectories) are available upon request from the authors.
