# Supplementary material for: Intrinsically dominant conformational diversity in PDZ1 within the tandem PDZ1–PDZ2 of human syntenin‐1 underlied by crystal structures
Source: Protein Sci. 2026 May 5;35(6):e70607. doi: 10.1002/pro.70607 (PMC13142097; doi:10.1002/pro.70607)
Supplement: Supplementary file 1 — Supplementary Figure S1. The chemical structures of the compounds used in crystallization experiments of human syntenin‐1 PDZ1‐PDZ2 tandem. Supplementary Figure S2. Average pairwise root mean square deviation (RMSD) for each residue calculated by pairwise comparison of crystal structures in the PDB database. The reference PDB ID: 1N99, 1OBZ, 1V1T, 1W9E, 1W9O, 1W9Q, 1YBO, 4Z33, 6R9H, 6RLC, 8AAI, 8AAK, 8AAO, 8AAP. Highlighted areas (red) are the sites of molecular fluctuations in the PDZ1 domain. (A): Average pairwise RMSD for each residue in the PDZ1 domain. (B): Average pairwise RMSD for each residue in the PDZ2 domain. The diagrams of the secondary structures are shown at the top of the panels. The red lines indicate the two loop regions of each PDZ domains. The high‐RMSD regions (>1.0 Å) on PDZ domain structures were highlighted as red. Supplementary Figure S3. Root mean square fluctuation (RMSF) for each residue calculated by MD calculations on the single PDZ1/PDZ2 domain (black) and the PDZ1–PDZ2 tandem structure (green). The S6_chain B structure was used for simulation. Highlighted areas show molecular fluctuations in the PDZ1 domain. (A): RMSF for each residue in the PDZ1 domain. (B): RMSF for each residue in the PDZ2 domain. The red lines indicate the two loop regions of each PDZ domains. The diagrams of the secondary structures are shown at the top of the panels. The high‐RMSF regions (>0.12 Å) on PDZ domain structures were highlighted as red. Supplementary Figure S4. 1H–15N Hetero‐NOE measurements by solution NMR. (A): Peak intensity ratio for each residue in the PDZ1 domain. (B): Peak intensity ratio for each residue in the PDZ2 domain. The diagrams of the secondary structures are shown at the top of the panels. The red lines indicate the two loop regions of each PDZ domains. The low peak intensity ratio regions (<0.75) on PDZ domain structures were highlighted as red. Supplementary Figure S5. Cluster analysis results with inter‐cluster distance values. (A [file PRO-35-e70607-s001.pdf]

Supplementary Materials

Intrinsically Dominant Conformational Diversity in PDZ1 within the Tandem PDZ1-PDZ2 of Human Syntenin-1 Underlined by Crystal Structures.

Natsuno Ando[1], Yuya Hanazono[2], Koya Sakuma[3], Nobutaka Numoto[2,4], Ryusei Hamajima[1], Takeshi Tenno[1,5], Atsunori Oshima[3,6,7], Nobutoshi Ito[2] and Hidekazu Hiroaki[1,5,7,*].

[1] Graduate School of Pharmaceutical Sciences, Nagoya University, Furocho, Chikusa, Nagoya, Aichi, Japan

[2] Laboratory of Structural Biology, Medical Research Institute, Tokyo Medical and Dental University (TMDU), 1-5-45, Yushima Bunkyo-ku, Tokyo, Japan

[3] Cellular and Structural Physiology Institute (CeSPi), Nagoya University, Furocho, Chikusa, Nagoya, Aichi, Japan

[4] International Center for Structural Biology, Research Institute for Interdisciplinary Science, Okayama University, Tsushima Naka 3-1-1, Kita, Okayama 700-8530, Japan.

[5] BeCellBar, LLC., Furocho, Chikusa, Nagoya, Aichi, Japan.

[6] Institute for Glyco-core Research (iGCORE), Tokai National Higher Education and Research System, Furocho, Chikusa, Nagoya, Aichi, Japan [正式名称?]

[7] Center for One Medicine Innovative Translational Research; COMIT, Nagoya University, Nagoya, Aichi, Japan

*** Correspondence:**

Hidekazu Hiroaki*

hiroaki.hidekazu.j7@f.mail.nagoya-u.ac.jp

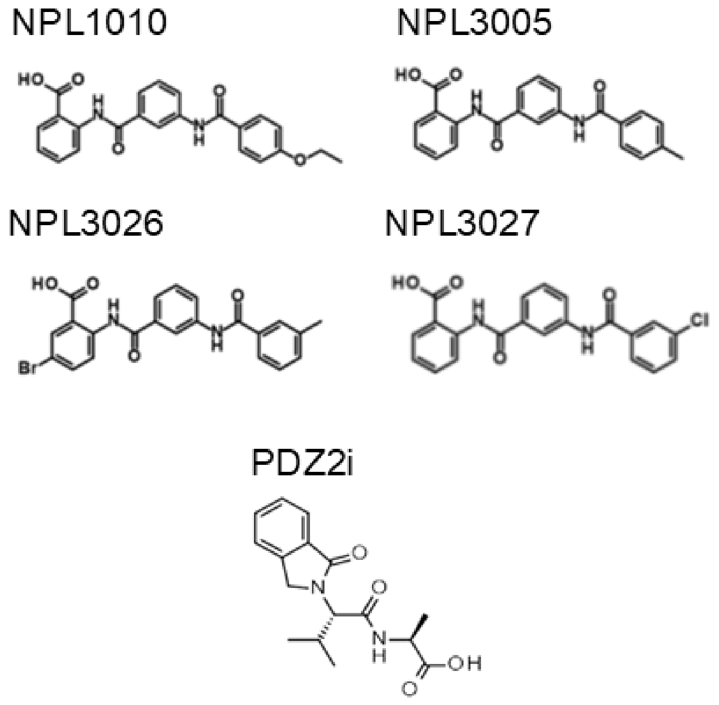

Supplementary Figure S1.

The chemical structures of the compounds used in crystallization experiments of human syntenin-1 PDZ1-PDZ2 tandem.

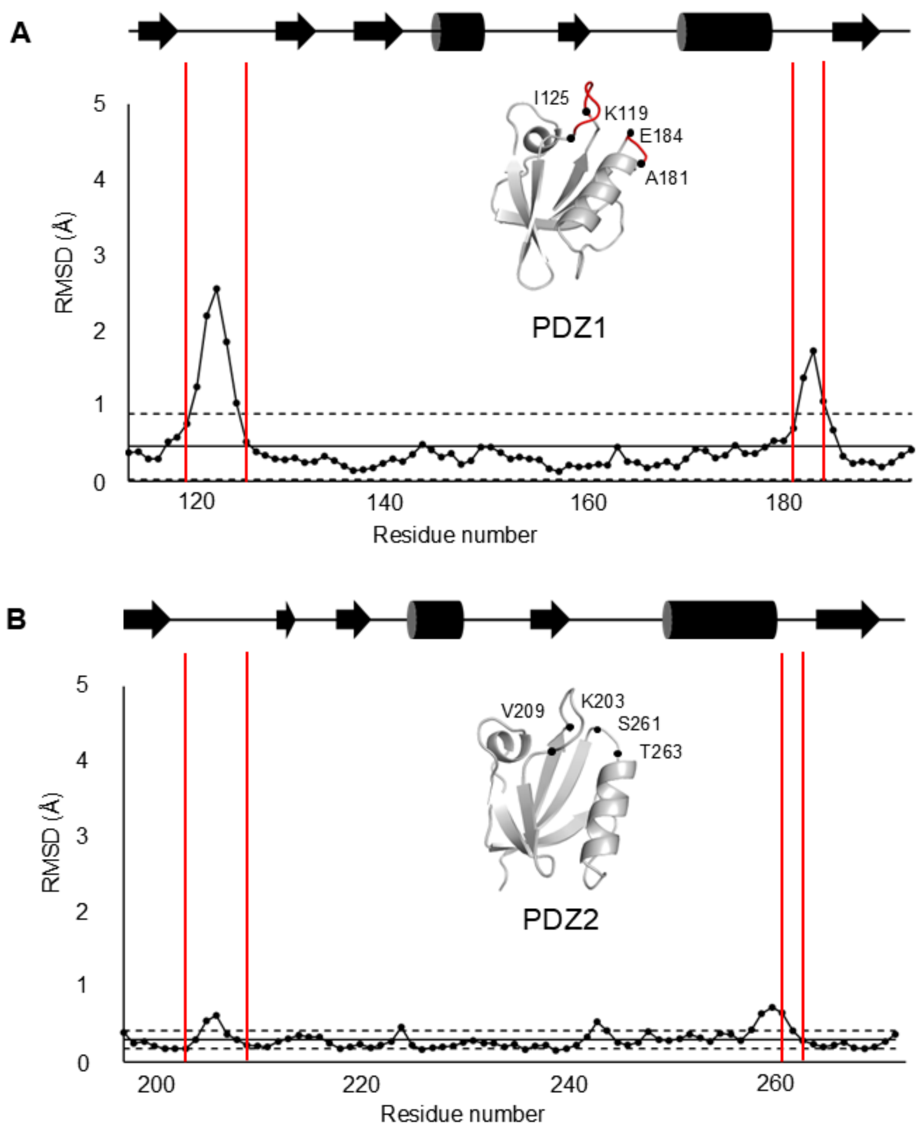

Supplementary Figure S2.

Average pairwise root mean square deviation (RMSD) for each residue calculated by pairwise comparison of crystal structures in the PDB database. The reference PDB ID: 1N99, 1OBZ, 1V1T, 1W9E, 1W9O, 1W9Q, 1YBO, 4Z33, 6R9H, 6RLC, 8AAI, 8AAK, 8AAO, 8AAP. Highlighted areas (red) are the sites of molecular fluctuations in the PDZ1 domain. A: Average pairwise RMSD for each residue in the PDZ1 domain. B: Average pairwise RMSD for each residue in the PDZ2 domain. The diagrams of the secondary structures are shown at the top of the panels. The red lines indicate the two loop regions of each PDZ domains. The high-RMSD regions (>1.0 Å) on PDZ domain structures were highlighted as red.

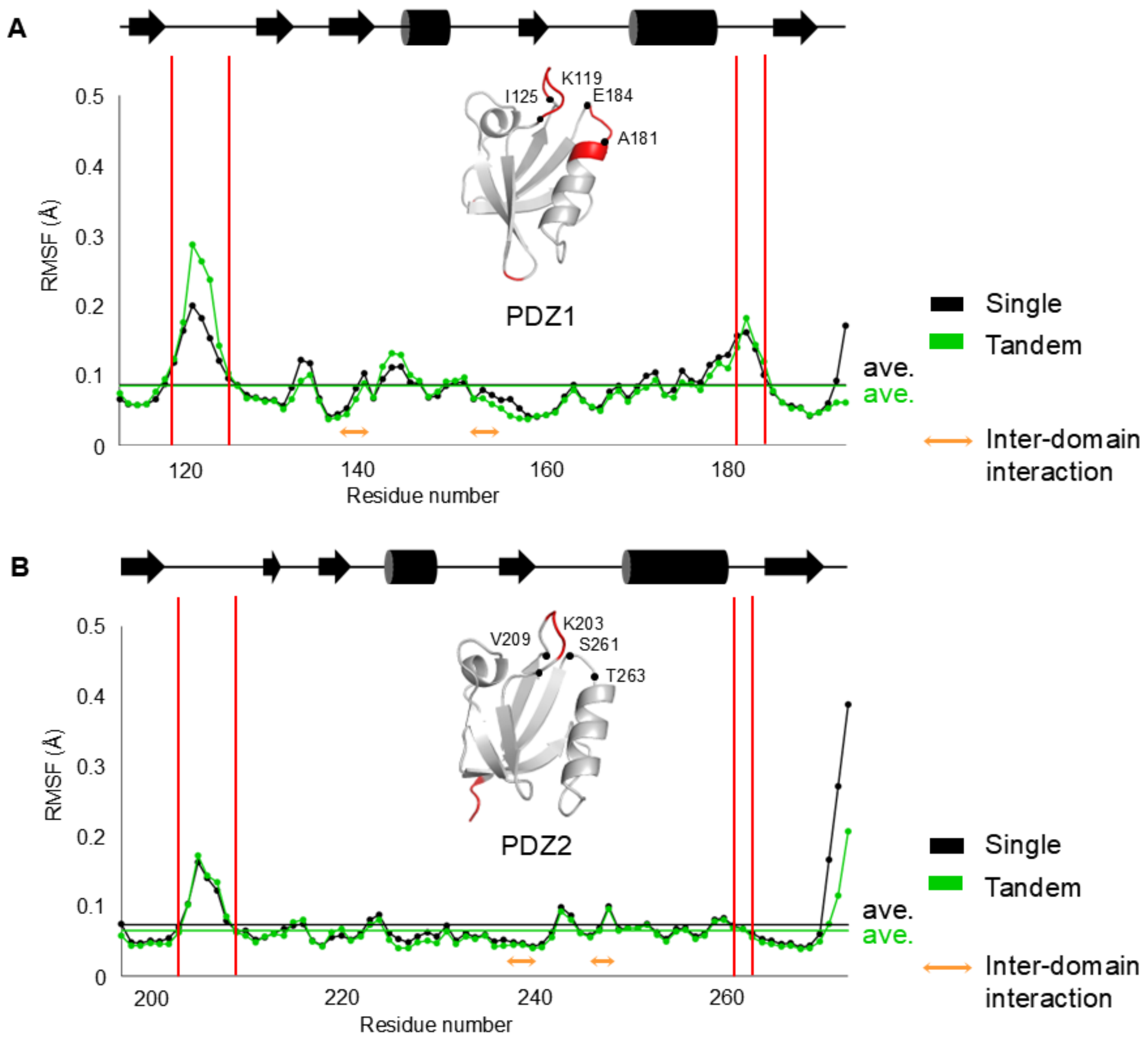

Supplementary Figure S3.

Root mean square fluctuation (RMSF) for each residue calculated by MD calculations on the single PDZ1/PDZ2 domain (black) and the PDZ1-PDZ2 tandem structure (green). The S6_chain B structure was used for simulation. Highlighted areas show molecular fluctuations in the PDZ1 domain. A: RMSF for each residue in the PDZ1 domain. B: RMSF for each residue in the PDZ2 domain. The red lines indicate the two loop regions of each PDZ domains. The diagrams of the secondary structures are shown at the top of the panels. The high-RMSF regions (>0.12 Å) on PDZ domain structures were highlighted as red.

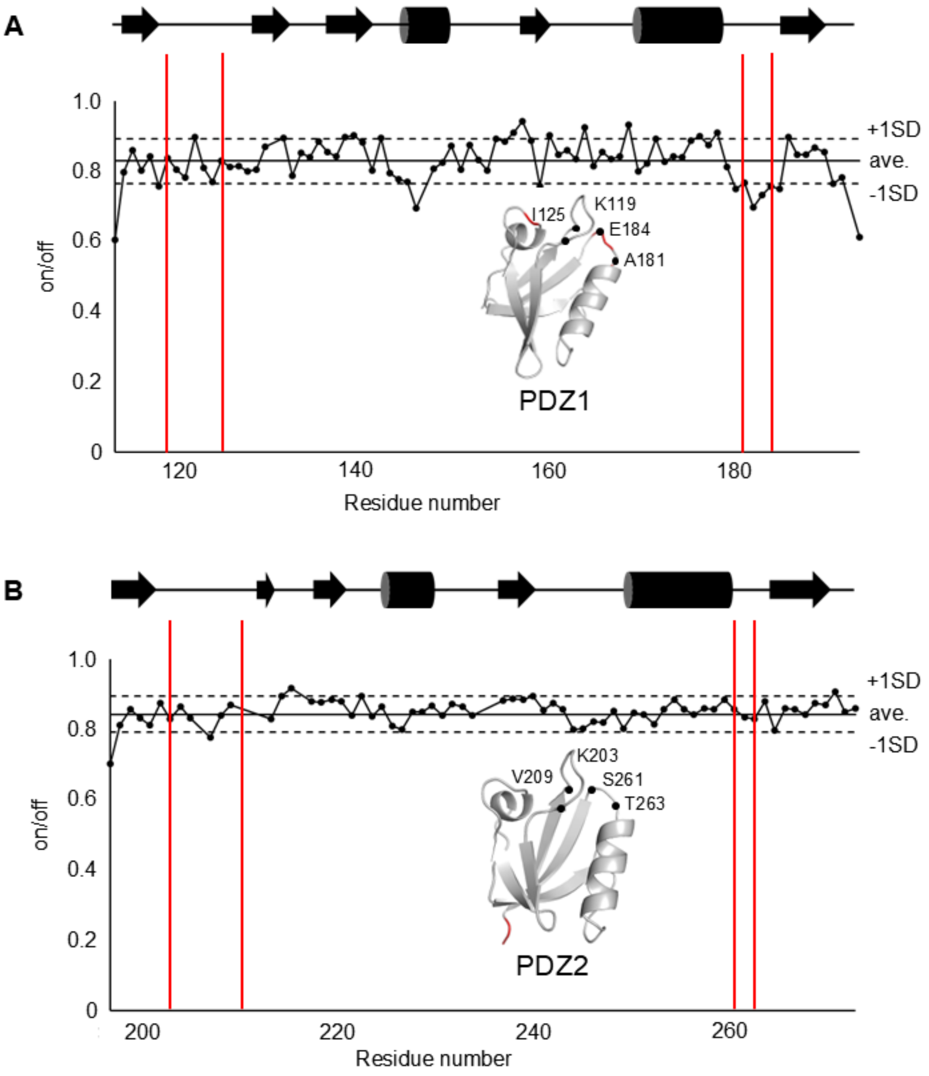

Supplementary Figure S4.

^{1}H-^{15}N Hetero-NOE measurements by solution NMR. A: Peak intensity ratio for each residue in the PDZ1 domain. B: Peak intensity ratio for each residue in the PDZ2 domain. The diagrams of the secondary structures are shown at the top of the panels. The red lines indicate the two loop regions of each PDZ domains. The low peak intensity ratio regions (<0.75) on PDZ domain structures were highlighted as red.

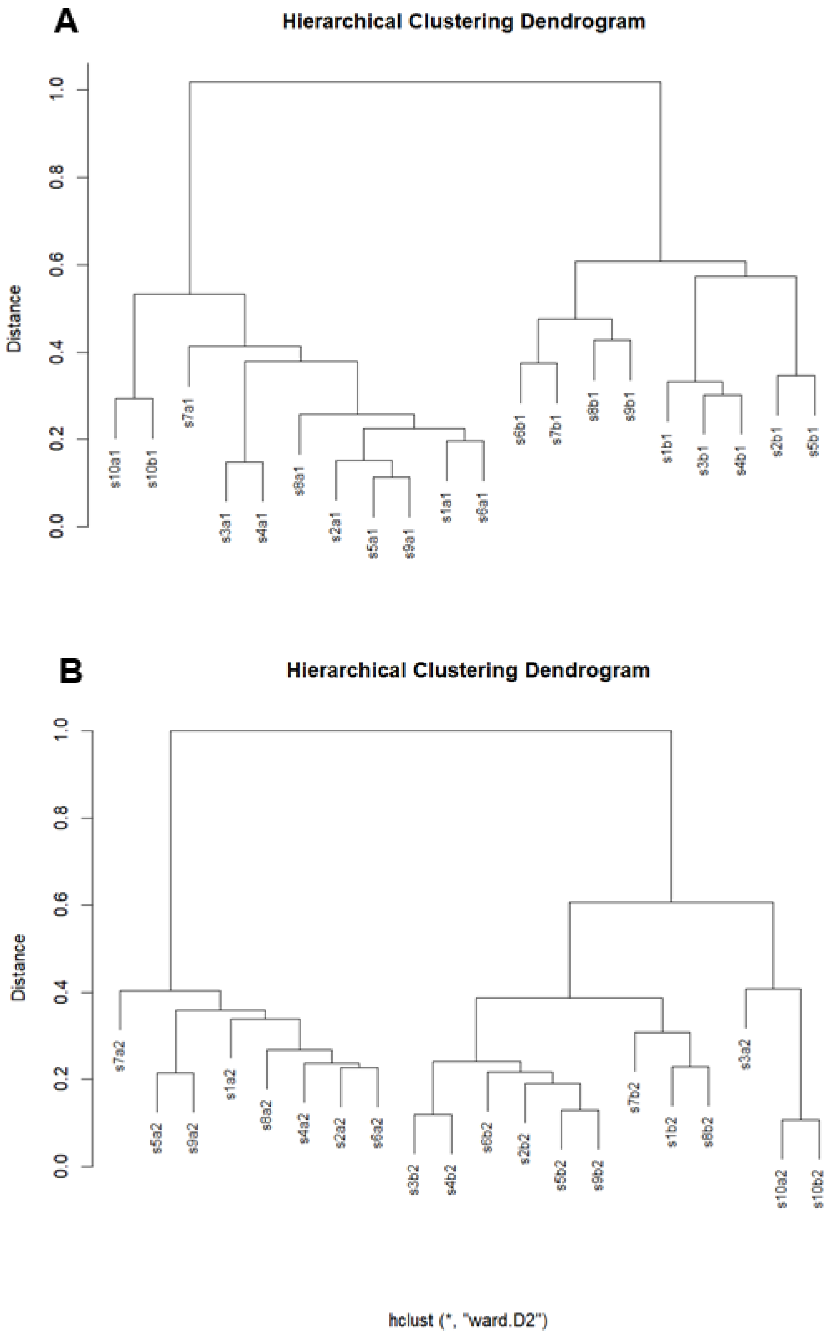

Supplementary Figure S5.

Cluster analysis results with inter-cluster distance values. A: Cluster analysis of PDZ1 domains. B: Cluster analysis of PDZ2 domains.

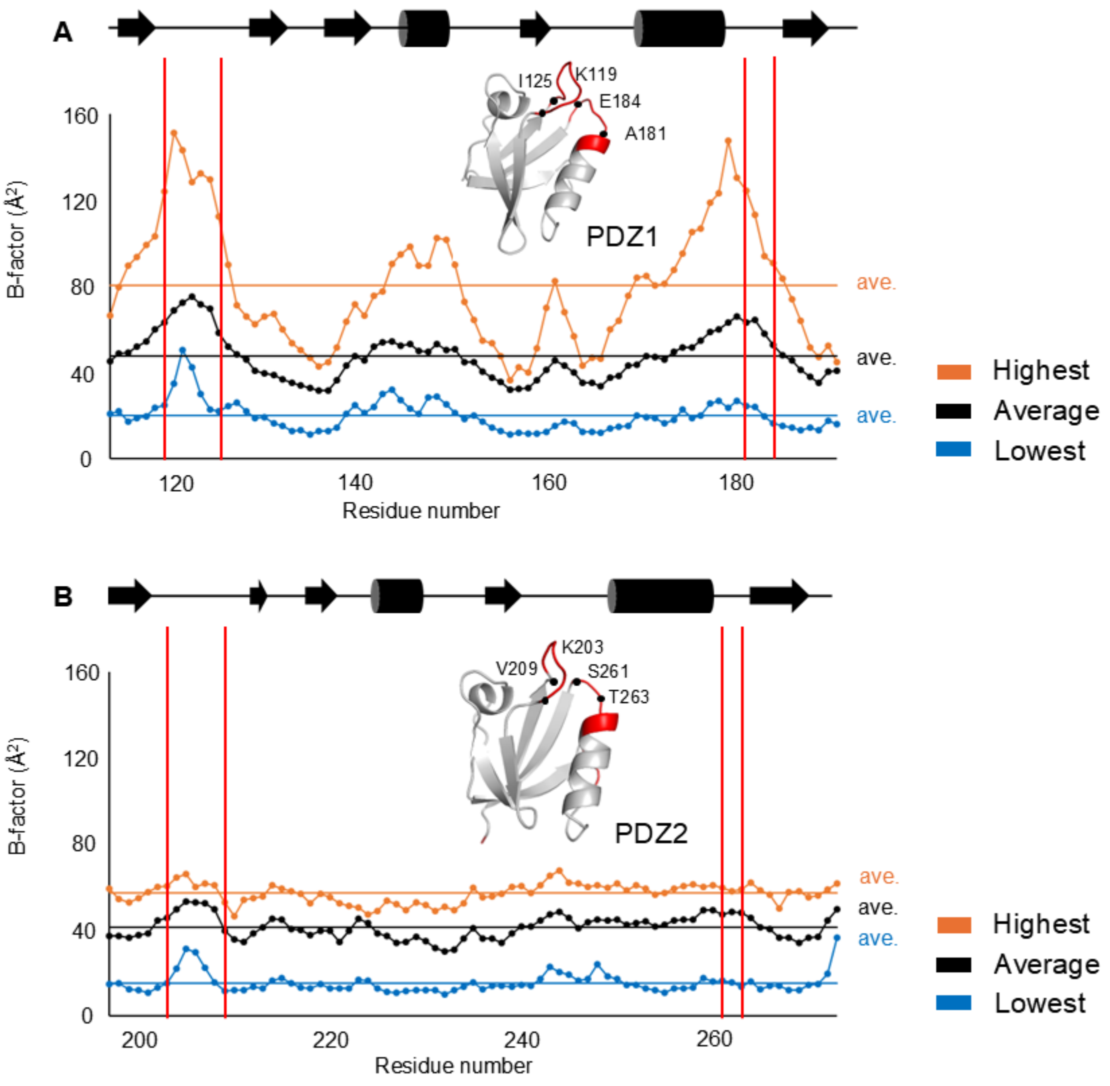

Supplementary Figure S6.

The B-factor values for the structure with the highest B-factor (orange), the average value across all structures (black), and the structure with the lowest B-factor (blue). A: The B-factor values for each residue in the PDZ1 domain. B: The B-factor values each residue in the PDZ2 domain. The diagrams of the secondary structures are shown at the top of the panels. The high B-factor regions (>ave.) on PDZ domain structures were highlighted as red.

Supplementary Table S1.

**Data Collection**			
	S1	S2	S3
Space group	P 31 2 1	P 31 2 1	P 31 2 1
Unit cell parameters (Å)	a = 56.95	a = 56.61	a = 56.88
	b = 56.95	b = 56.61	b = 56.88
	c = 150.36	c = 150.72	c = 155.85
Wavelength (Å)	0.98	0.98	0.98
Resolution (Å)	46.87-2.09	49.02-1.81	46.97-2.02
	(2.15-2.09)	(1.85-1.81)	(2.07-2.02)
Total number of unique reflections	17,500 (1,324)	26,422 (1,524)	19,997 (1,447)
Completeness (%)	100 (100)	100 (100)	100 (100)
Average I/σ	14.4 (2.2)	24.4 (2.2)	15.6 (2.3)
Redundancy	9.6 (9.9)	9.8 (10.3)	9.8 (10.2)
R_{merge} (%)	0.099 (1.173)	0.054 (1.188)	0.123 (1.176)
$CC_{1/2}$(%)	(0.731)	(0.751)	(0.792)
**Refinement**			
R_{work}/R_{free}	0.277/0.336	0.259/0.299	0.231/0.274
Number of atoms			
Protein	2,530	2,526	2,540
Water	24	83	95
Average B factor (Å^2)	58.0	44.0	40.0
Root mean square deviations			
Bond length (Å)	0.12	0.19	0.10
Bond angles (°)	0.31	0.37	0.29
Ramachandran analysis			
Favored (%)	94.5	96.0	95.1
Allowed (%)	5.2	3.4	4.6
Outliers (%)	0.3	0.6	0.3

(continued)

	S4	S5	S6
**Data Collection**			
Space group	P 31 2 1	P 31 2 1	P 31 2 1
Unit cell parameters (Å)	a = 57.02	a = 56.68	a = 56.90
	b = 57.02	b = 56.68	b = 56.90
	c = 154.75	c = 149.56	c = 151.72
Wavelength (Å)	0.98	0.98	0.98
Resolution (Å)	47.04-2.05	49.08-1.85	46.87-1.86
	(2.11-2.05)	(1.89-1.85)	(1.90-1.86)
Total number of unique reflections	19,132 (1,481)	24,661 (1,525)	24,787 (1,514)
Completeness (%)	100 (100)	100 (100)	100 (100)
Average I/σ	20.8 (2.3)	26.6 (2.3)	22.7 (2.2)
Redundancy	9.7 (10.2)	9.7 (10.4)	9.8 (10.4)
R_{merge} (%)	0.083 (1.157)	0.047 (1.144)	0.060 (1.100)
$CC_{1/2}$(%)	(0.772)	(0.798)	(0.785)
**Refinement**			
R_{work}/R_{free}	0.243/0.294	0.271/0.321	0.249/0.297
Number of atoms			
Protein	2,540	2,530	2,518
Water	81	60	47
Average B factor (Å^2)	49.0	45.0	44.0
Root mean square deviations			
Bond length (Å)	0.10	0.30	0.39
Bond angles (°)	0.28	0.57	0.59
Ramachandran analysis			
Favored (%)	95.1	95.7	97.2
Allowed (%)	4.3	4.3	2.8
Outliers (%)	0.6	0	0

(continued)

**Data Collection**				
	S7	S8	S9	S10
Space group	P 31 2 1	P 31 2 1	P31 2 1	P 1 21 1
Unit cell parameters (Å)	a = 56.88	a = 56.97	a = 56.96	a = 59.87
	b = 56.88	b = 56.97	b = 56.96	b = 30.09
	c = 149.20	c = 152.03	c = 150.47	c = 91.06
Wavelength (Å)	0.98	0.98	0.98	1
Resolution (Å)	46.78-2.15	49.34-2.31	49.33-1.90	49.68-1.40
	(2.22-2.15)	(2.39-2.31)	(1.94-1.90)	(1.42-1.40)
Total number of unique reflections	15,951 (1,339)	13,240 (1,281)	23,132 (1,412)	64,791 (3,233)
Completeness (%)	100 (100)	100 (100)	100 (99.9)	100 (100)
Average I/σ	11.3 (2.1)	9.9 (2.1)	20.6 (2.1)	15.2 (3.1)
Redundancy	9.5 (9.6)	9.5 (9.4)	9.7 (10.4)	16.2 (16.3)
R_{merge} (%)	0.122 (1.133)	0.151 (1.124)	0.061 (1.292)	0.082 (1.153)
$CC_{1/2}$(%)	(0.736)	(0.724)	(0.770)	(0.950)
**Refinement**				
R_{work}/R_{free}	0.304/0.361	0.245/0.313	0.264/0.302	0.185/0.220
Number of atoms				
Protein	2,518	2,517	2,518	2,671
Water	13	18	33	360
Average B factor (Å^2)	60.0	54.0	53.0	27.0
Root mean square deviations				
Bond length (Å)	0.14	0.52	0.25	0.32
Bond angles (°)	0.34	0.71	0.49	0.51
Ramachandran analysis				
Favored (%)	96.6	91.4	95.1	98.0
Allowed (%)	3.4	8.0	4.6	1.1
Outliers (%)	0	0.6	0.3	0.8
